# Draft Genome Sequence of *Burkholderia cordobensis* Type Strain LMG 27620, Isolated from Agricultural Soils in Argentina

**DOI:** 10.1128/genomeA.01238-15

**Published:** 2015-10-22

**Authors:** Walter Omar Draghi, Ulises M. Mancini Villagra, Luis Gabriel Wall, Angeles Zorreguieta

**Affiliations:** a Fundación Instituto Leloir, IIBBA CONICET, Buenos Aires, Argentina; b Facultad de Ciencias Exactas, Instituto de Biotecnología y Biología Molecular (IBBM), Universidad Nacional de la Plata-CCT, CONICET, La Plata, Argentina; c Departamento de Ciência y Tecnología, Universidad Nacional de Quilmes, Bernal, Argentina

## Abstract

Bacteria of the genus *Burkholderia* are commonly found in diverse ecological niches in nature. We report here the draft genome sequence of *Burkholderia cordobensis* type strain LMG 27620, isolated from agricultural soil in Córdoba, Argentina. This strain harbors several genes involved in chitin utilization and phenol degradation, which make it an interesting candidate for biocontrol purposes and xenobiotic degradation in polluted environments.

## GENOME ANNOUNCEMENT

The *Burkholderia* genus comprises >80 validly described species, isolated from diverse niches in nature. Species within the *Burkholderia cepacia* complex (Bcc) are mainly recognized as opportunistic pathogens of cystic fibrosis or immunocompromised patients (1, 2). However, several *Burkholderia* species have also been isolated from diverse ecological niches, such as agricultural soils, root nodules, the gut microbiota of insects, contaminated soils, endophytes of leaves or plant roots, and the interior of spores or fungi mycelia, indicating the wide versatility of this genus (3–12).

The whole-genome shotgun sequencing of *Burkholderia cordobensis* type strain LMG 27620, isolated from an agricultural soil in Córdoba Province, Argentina, was performed (13). Genomic DNA was extracted from tryptone soybean agar cultures using the method of Pitcher et al. (14). The genome was sequenced by Macrogen, Inc. (Geumcheon-gu, Seoul, Republic of Korea) using an Illumina HiSeq 2000 sequencing platform. A total of 1,002,545,392 reads were obtained, with a G+C content of 62.5%. Paired-end reads (2 × 100 bp) were trimmed and assembled *de novo* using the A5 pipeline, obtaining 69 scaffolds (longest scaffold, 1,508,897 bp; *N*_50_, 70,128 bp) with 105× coverage (15). Scaffolds were ordered in Mauve (16) with the genome of *B. cordobensis* YI23 as a reference. The genome consists of 9,044,501 bp. Automatic gene prediction and functional annotation were carried out by using the RAST server (17), revealing 8,556 protein-coding sequences, 55 tRNAs, 3 rRNAs, and 43 ribosomal proteins.

Several *Burkholderia* species are able to degrade xenobiotic compounds. In fact, phylogenetically closely related strains, such as *B. cordobensis* YI23, *Burkholderia* sp. SJ98, *Burkholderia jiangsuensis* MP-1, and *Burkholderia zhejiangensis* OP-1, have been isolated from polluted environments (18–20). It was previously shown that *B. cordobensis* YI23 and *Burkholderia* sp. SJ98 share a gene cluster involved in chemotaxis toward compounds that they degrade, such as chloronitroaromatic compounds (21, 22). *B. cordobensis* LMG 27620 also harbors the same cluster of chemotaxis genes. In addition, several genes of biotechnological interest were detected. Phenol monooxygenase genes and its regulator genes and paraquat-induced proteins were also found in the LMG 27620 genome. In addition, genes involved in biocontrol process were present, as chitinases and *N*-acetylglucosamine utilization were observed. Overall, the evidence presented here turns this strain into a biotechnological potential microorganism to be involved in pest biological control and biodegradation of pollutants in contaminated environments.

### Nucleotide sequence accession numbers. 

This whole-genome shotgun project has been deposited at DDBJ/EMBL/GenBank under the accession no. LGRC00000000. The version described in this paper is version LGRC01000000.
